# Two Cases of Poisoning by the Fruit of the Podophyllum Peltatum, or Mayapple

**Published:** 1863-08

**Authors:** D. C. Owen

**Affiliations:** Houston, Ill.


ARTICLE XXI.

### TWO CASES OF POISONING BY THE FRUIT OF THE PODOPHYLLUM PELTATUM OR MAYAPPLE.

BY DR. D. C. OWEN, of Houston, Ill.

On the 4th of August, 1860, I was called upon to visit two children of H. C., who, the messenger said, were vomiting themselves to death. I enquired what the children had been eating or taking; he replied, that the parents thought the vomiting was caused by Mayapples, which the children had eaten in the morning.

I drove briskly on, and arrived at the place in, perhaps, half an hour, where I found two very pretty little girls, aged, respectively, six and eight years, stretched upon their beds, bathed in cold perspiration; their faces pale as corpses; eyes sunken in their orbits; noses pinched; pulse very weak, and scarcely perceptible at the wrist; great thirst; and the stomach contracting so hard and rapidly, in efforts to vomit, that the wrenching pain would cause them to utter sharp screams, one after another, for five minutes at a time. I was told, that the vomiting had been going on for the last four hours, almost without intermission, it being now 12 M.

On examining the matters thrown from the stomach, I found them to consist, for the most part, of seeds, pulp, and membranous covering of the ripe Mayapple, having the peculiar odor of that fruit. I asked them, if they had eaten the rinds of the apples? They said, no; but they had used their teeth to rupture the rinds.

I informed the parents that the recovery of the oldest child was not to be expected, owing to the prostration already produced; but that the chances for the younger were much better. I gave the oldest 1 gr. sulph. morphine, covered the epigastrium and entire abdomen, which was tympanitic and very tender, with a blister; invited the blood to the extremities with hot flannels and sinapisms; gave carbonic acid water, in small quantities, to allay thirst; and, as the bowels had not been moved since the vomiting begun, ordered an enema of castor oil and molasses in warm water. To the younger I gave, perhaps, $\frac{2}{3}$ gr. morphine, and ordered the same course as for the other, except that for the blister I substituted mustard. They were now kept as quiet as possible for, perhaps, an hour and a-half, when the younger child was sufficiently under the influence of the narcotic to fall into a quiet sleep. The oldest now vomited again bilious matter mixed with blood,—the bile dark green and very thick, the blood dark and coagulated. She complained frequently of burning sensation in the throat. Finding that the injections had failed to move the bowels, I gave Hydrargyri sub. mur.,.........................gr. xij.
Morphine sulph.,..................................gr. j.

They were now both getting warm extremities, the youngest still sleeping. I left powders of morphine and camphor, one of which was to be given after each act of vomiting, should it commence again, or after each stool, should the bowels act too freely. I directed the parents to do everything in their power to maintain the proper amount of heat in the extremities; and, if the means they were then using were not sufficient to keep up the warmth of the body, to use brandy by enema. For the youngest, I left similar powders and directions, should she require them.

Called, August 5th, at 7 A.M.—Found the oldest past all hope,—the eyes glazed and motionless, the death-rattle in the throat, the abdomen swelled almost to bursting, and the under jaw fallen. At half-past eight o'clock she expired. I learned that she had continued to vomit thick bile, with more or less blood mixed with it, about every hour through the night. The youngest child was considerably better, although there was considerable tenderness over the stomach, to remove which I applied a blister; gave Dover's powder to keep her quiet, slippery-elm water for drink, kept the bowels solvent with sulphate of magnesia; and, by the morning of the 8th, she was convalescent.

I have endeavored to give a simple statement of the facts in the above cases, as they occurred; and I will decline making any comments, more than to say, that, after seeing the deleterious effects of the Mayapple in these cases, I would not recommend them as being the healthiest article of diet that we can procure; and I have also kept an eye single on the action of podophylline.

